# Silsesquioxanes in the Cosmetics Industry—Applications and Perspectives †

**DOI:** 10.3390/ma15031126

**Published:** 2022-01-31

**Authors:** Anna Olejnik, Bogna Sztorch, Dariusz Brząkalski, Robert E. Przekop

**Affiliations:** 1 Faculty of Chemistry, Adam Mickiewicz University in Poznań, Uniwersytetu Poznańskiego 8, 61-614 Poznań, Poland; dariusz.brzakalski@amu.edu.pl; 2 Centre for Advanced Technologies, Adam Mickiewicz University in Poznań, Uniwersytetu Poznańskiego 10, 61-614 Poznań, Poland; bogna.sztorch@amu.edu.pl (B.S.); rprzekop@amu.edu.pl (R.E.P.)

**Keywords:** silsesquioxanes, personal care, cosmetics, industry, POSS, polysilsesquioxanes

## Abstract

The rising demand for innovative and sophisticated personal care products is a driving factor for manufacturers to obtain new formulations that will fulfill the customers’ preferences. In recent years, silsesquioxanes have attracted the attention of the cosmetics industry. These compounds have been proposed to be used in novel cosmetic formulations as emollient, dispersant, and viscosity modifiers. Therefore, this publication aims to review the main important aspects of polyhedral oligosilsesquioxanes as ingredients of personal care formulations, taking into consideration different types of products. The methods of obtaining these compounds were also presented. Additionally, the detailed analysis of patents dedicated to the application of silsesquioxanes in cosmetic formulations was also performed.

## 1. Introduction

The global cosmetics market is constantly growing each year, and it is expected to reach $429.8 billion in 2022 according to Allied Market Research [1]. The rising demand for innovative and sophisticated personal care products is a driving factor for manufacturers to obtain new formulations that will fulfill the customers’ preferences. Different aspects should be taken into consideration while developing novel cosmetic products such as appearance, rheology, consistency, ease of removal, and functionality (sun-screening, antioxidant, and moisturizing effects). In recent years, silicones have become more and more significant in modern cosmetic products. However, it should be mentioned that silicones were first applied in personal care formulations in the 1940s [2]. Since that time, the rapid increase in the production of cosmetics containing this class of compounds has been observed [3,4,5]. The term “silicone” that is often used in the cosmetic industry refers to a great diversity of ingredients that have different solubility and distinctive features. They are classified as follows:

- Silanes
- Hydroxyl-containing compounds (silanols)
- Cyclic dimethyl siloxanes (cyclomethicones)
- Linear Polysiloxanes (trimethylsiloxy end-capped, non-trimethylsiloxy end-capped)
- Silicates (inorganic)
- Copolymers and silsesquioxanes (SSQs) [6].

These ingredients have found applications in personal care products due to their unique properties. They exhibit the ability to reduce surface tension after their incorporation into cosmetic products. This property is especially important to facilitate the spreadability of formulations dedicated both to hair and skin. It was reported by Lardy et al. [7] and Budiasih et al. [8] that the lower the surface tension, the higher the spreadability of the formulation applied on the skin. The surface tension of silicones is ca. 20 dynes/cm^2^, which is significantly different from the surface tension of water (76 dynes/cm^2^) and oils (32 dynes/cm^2^) [6]. 

Many myths and misconceptions about silicones have appeared in the cosmetic world recently. It is believed that these compounds clog the pores and aggravate acne. However, based on scientific studies, silicones are resistant to oxidation and are not occlusive. Therefore, they are not able to clog the pores. Furthermore, they do not intensify acne caused by *Propionibacterium acnes* [9]. Adams et al. proved that silicone oil exhibited no effect on bacterial growth [10]. On the other hand, silicones can form a film on the surface of the skin that may influence the penetration of active compounds into the *stratum corneum* [11]. It is claimed that silicones are irritating. However, they are neutral to the skin. On the contrary, silicones reduce skin irritations [6]; therefore, they are used in soothing preparations. For many years, they have been applied in pharmaceutical forms. Dimethicone, silicone oil, is an ingredient of commonly known preparations against flatulence and colic [12]. It is stated that silicones accumulate on the skin. Some silicones (silicone resins and silicone acrylates) create a film on the skin surface, but due to the unique structure of their molecules, this film is fully permeable to water vapor and oxygen [13,14,15]. As a result, the ideal conditions for skin regeneration, wound healing, and reduction of scars are created [15,16]. It has been proven that this environment is appropriate for the synthesis of collagen [17].

In recent years, polyhedral oligosilsesquioxanes (POSS/SSQs) have attracted the attention of the cosmetic industry. These compounds have been proposed to be used in novel cosmetic formulations as emollient, dispersant, and viscosity modifiers [18,19,20]. SSQs possess a structural resemblance to silicones, as their backbone is based on Si–O–Si bonds, due to which they are sometimes called cage siloxanes. It should be highlighted that the simplest form of POSS with hydrogen attached to the cage has limited applications in cosmetics due to its low stability and solubility in formulations [21]. The attachment of organic groups to the silica-like core makes POSS more soluble [22] and chemically stable and thus more appropriate as additives to skincare products. The introduction of either hydrophilic or hydrophobic groups can be responsible for changes in the lipophilic balance. The “R” groups used as substituents in the corners of the POSS cage include quaternary nitrogen, zwitterionic compounds, and different functional organic groups [23]. Thus, a large number of compounds with various properties can be obtained [24], which have the potential to be used in personal care formulations. The functions of POSS in cosmetics depend on the type of product. The presence of certain POSS in the formulation can provide advantages in terms of compatibility or even solubility. To the best of our knowledge, there is no paper describing the application of POSS derivatives in cosmetics. Furthermore, the detailed analysis of patents dedicated to the application of silsesquioxanes in cosmetic formulations has not been performed before. Therefore, this publication aims to review the main important aspects of polyhedral oligosilsesquioxanes as ingredients of personal care formulations, taking into consideration different types of products.

## 2. Data Sources and Search Model for the Study

For a literature search covering silsesquioxane chemistry, structure, and nomenclature, the Scopus database and Google Scholar were used, and “silsesquioxane”, “POSS”, “cosmetics”, and “personal care” keywords were used. For the Scopus database, special attention was paid to review type documents.

For the patent search, “silsesquioxane”, “cosmetics”, and “personal care” keywords were used. The scope of the search was set to throughout all the years, but for clarity, only the results from 1990–2021 were presented in the form of a figure in Section 4 (Literature review on patent analysis regarding silsesquioxanes in cosmetics), as before that time, only single results were available, which would affect the readability of the graph.

## 3. Structure of Molecular and Macromolecular POSS Derivatives and Their Polymeric Hybrids

Polyhedral oligosilsesquioxanes, also known as polyhedral oligomeric silsesquioxanes (POSS and SSQs), represent a group of compounds that are more frequently applied in cosmetic products. They belong to silicon-containing substances, in which each Si atom is connected with three oxygen atoms. The fourth position on the silicon atom is combined with either hydrogen or organic groups (R). In simple terms, POSS are organosilicon compounds that consist of a polyhedral siloxane core and R groups that could be the same or different on each of the corners [25]. POSS were first obtained in the late 1940s, but it took over twenty years for these molecules to obtain the attention of scientists from all over the world. They possess a 3D multi-armed structure and therefore exhibit unique properties compared to traditional silicones [26]. Moreover, POSS are environmentally friendly, recyclable, and biocompatible. Additionally, they can be easily modified by the introduction of different organic functional groups to the silica-like core, which makes POSS even more attractive for industrial applications. The derivatives of POSS are odorless and nonvolatile under normal conditions. Moreover, they are environmentally friendly. They were commercialized by Hybrid Plastics Inc. and found their first application as plastics modifiers. However, they were also studied in electronic and optical fields [25]. Moreover, POSS can be appropriate compounds for medical and cosmetic applications due to their non-toxicity and cytocompatibility [18,27].

### 3.1. POSS Structure and Nomenclature

**Figure 1:**
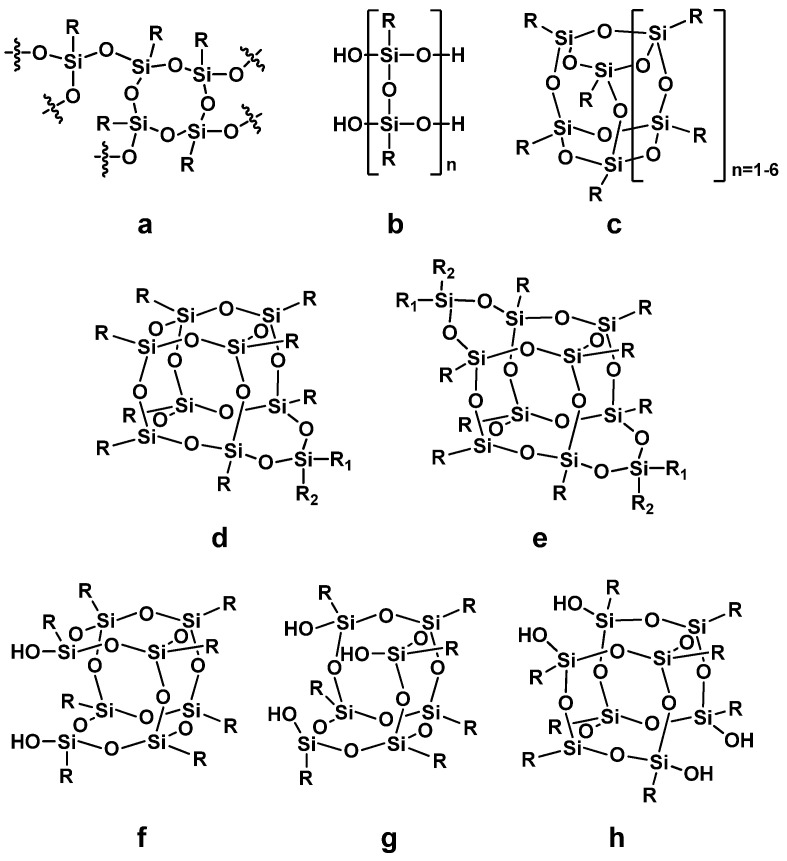
General scheme of different silsesquioxane categories: (**a**) random (T-resin, polysilsesquioxane); (**b**) ladder; (**c**) cage T series; (**d**) homosilsesquioxane; (**e**) double-decker; (**f**) disilanol; (**g**) trisilanol; (**h**) tetrasilanol.

Silsesquioxanes are, by definition, compounds following the general formula [RSiO_3/2_]_n_. However, this formula does not represent the high variety of structures that may be formed upon their synthesis under different reaction conditions. Silsesquioxanes may form random, polymeric networks, usually called polysilsesquioxanes, alternatively known under the name “T-resins” (Figure 1a) [19]. These are either resinous or solid materials, usually amorphous, and may be supplied or synthesized as (nano)particles of the desired size. In some cases, ordered ladder structures of different molar masses are obtained (Figure 1b). Under controlled conditions, well-defined molecular structures (cage structures) may be obtained (Figure 1c), usually denoted as T_n_, where n is an even number in the range of 6–16 (e.g., T_8_, T_10_, and T_12_, the other structures being less likely to form, the reaction mixture showing a tendency towards polymeric products formation of smaller chain strains, either Figure 1a or Figure 1b). Silsesquioxanes are composed of T siloxane unit, and their empirical formula is as follows [RSiO_3/2_]_n_. Often, a simplified name is used for silsesquioxanes bearing the same organic group in all corners (e.g., phenylsilsesquixane when R is phenyl); however, the cage size (the number of T units) should always be also mentioned (e.g., octaphenylsilsesquioxane for Ph_8_Si_8_O_12_).

Furthermore, different molecular hybrids are also known, such as homosilsesquioxanes (enlarged cage, Figure 1d,e, of which 1e is known as double-decker silsesquioxane, DDSQ) or incompletely condensed cages (Figure 1f–h). There is, however, a common misconception in the understanding of these compounds’ structure and behavior. Polysilsesquioxanes are indeed high molecular weight polymeric structures, which, under proper conditions, may be processed into or formed as nanoparticles. On the other hand, it may be encountered in the literature reports where even the molecular POSS compounds are referred to as “nanoparticles”, despite having low, well-defined molecular mass and fully dissolving in organic solvents. This misinterpretation may come from two sources. One is calculations and measurements, which show that a single POSS molecule is, depending on the organic substituents, from 0.8 nm up to several nanometers in diameter. The second is the fact that many POSS compounds are crystalline and, upon mixing with some poorly compatible matrices (e.g., polymers and mineral fillers), they tend to self-segregate, forming crystalline or polycrystalline particulate agglomerates visible using scanning electron microscopy (SEM) or transmission electron microscopy (TEM) [28]. However, despite large molecule size, these compounds should not be considered nanoparticles, but rather the matrix they are introduced into, together with processing methods applied, will decide on the level of components mixing obtained (either micro- or nanoagglomeration, or dispersion/dissolution on a molecular level, with the formation of liquid or solid solution).

**Figure 2:**
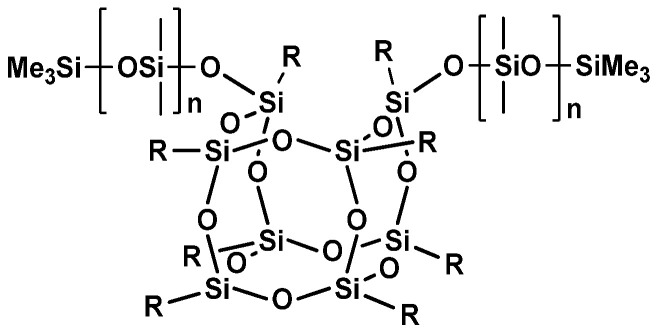
An exemplary structure of silsesquioxane–PDMS hybrid polymer.

Additionally, hybrid structures of silsesquioxanes and poly(dimethylsiloxanes) (PDMS) have been obtained and applied as additives for cosmetic products. In this case, a silsesquioxane molecule serves as a chain extender or branching node, modifying the rheological or emulsifying properties of the obtained hybrid (Figure 2).

Silsesquioxanes are composed of T siloxane unit, and their empirical formula is as follows: RSiO_3/2_. When the R group is not methyl, the prefix should be added (e.g., phenylsilsesquixane when R is phenyl).

### 3.2. Methods of Synthesis of Silsesquioxanes 

The synthesis of octosubstituted silsesquioxanes often proceeds in multi-stage reactions with a low yield of final products, resulting in a product mixture consisting of fully and incompletely condensed silsesquioxanes. The most common procedure for the preparation of silsesquioxanes containing the T8 cage structure is the hydrolysis of the appropriate trichloro- or trialkoxysilanes followed by condensation [29] and the corner capping method, i.e., obtaining bifunctional silsesquioxanes, mainly of R_7_R’Si_8_O_12_ type, by closing the corner of not fully condensed silsesquioxanes with chloro- or alkoxysilane [30]. Obtained derivatives can be functionalized in different catalytic or non-catalytic reactions (e.g., hydrosilylation, metathesis, silylative coupling, nucleophilic substitution, condensation) [31,32].

#### 3.2.1. Hydrolitic (olygo)condensation

**Figure 3:**
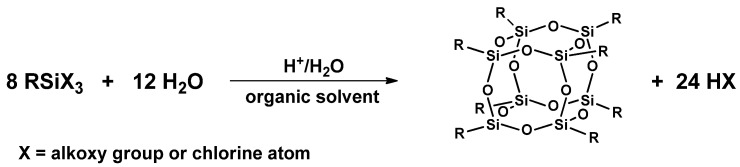
General procedure for the synthesis of cage silsesquioxanes via hydrolytic condensation of organosilanes.

The hydrolytic condensation method (Figure 3) is used to obtain fully condensed derivatives containing reactive functional groups in their structure, e.g., H, halogen, vinyl, NH_2_. The first step of the reaction is the hydrolysis of the appropriate trialkoxy- or trichlorosilane (RSiX_3_ (where X is a reactive group, susceptible to hydrolysis, e.g., halogen, alkoxy, or acyloxy) in the presence of water to form silanols. This is catalyzed by acids, bases, or nucleophiles (HCl, KOH, fluoride anion) [33]. In the next stage, the condensation reaction of silanols formed in situ in an acidic or basic conditions [32]. The course of the process and the structure of the resulting silsesquioxane are impacted by many factors, such as the nature of the functional groups, the type of hydrolysis catalyst, the selection of an appropriate solvent, and process conditions (temperature, pH, silicon precursor concentration, silane addition rate, temperature, and reaction time) [31,34]. The method of hydrolysis and condensation usually runs with low yields of products due to non-selective synthesis conditions [35].

#### 3.2.2. Corner Capping

**Figure 4:**
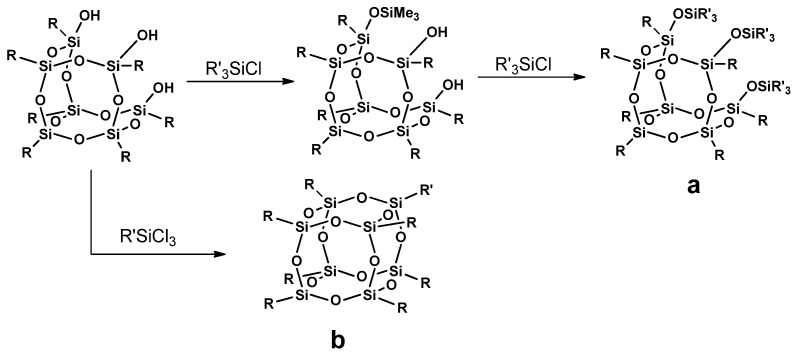
A procedure of corner capping of open-cage silsesquioxane trisilanol; (**a**)—a product of silanol groups capping with monochlorosilanes; (**b**)—a cage T_8_ product of actual corner capping with trichlorosilane.

Another popular method used in the synthesis of cage silsesquioxanes is corner capping. This reaction undergoes the silsesquioxanes of the incompletely condensed structure T_7_-triol (eg., Trisilanol POSS, Figure 4), in which “cage closing” takes place by reaction with an appropriate alkoxy- or chlorosilane in the presence of a basic catalyst, e.g., NEt_4_OH. This method allows obtaining derivatives containing the desired functional groups at the corner, affecting the changes in the physicochemical properties of the new derivative [36]. 

#### 3.2.3. Synthesis of Silsesquioxane Resins/Polysilsesquioxanes

**Figure 5:**
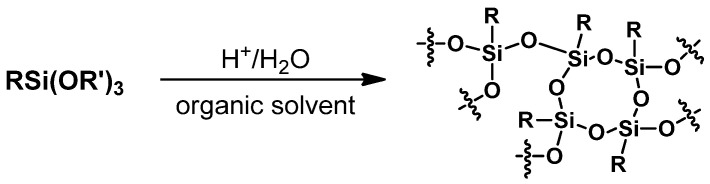
Synthesis of silsesquioxanes with disordered structure.

This type of silsesquioxanes is of particular importance in chemistry and the production of cosmetics; they improve the dispersion of pigments and are used in hair care and styling preparations due to their high resistance to weather conditions [6]. Silsesquioxane resins can be obtained by strong acid or base-catalyzed hydrolysis and polycondensation of alkoxysilanes with the possibility of further cross-linking with an organic matrix (Figure 5) [37]. In general, the preparation methods for obtaining polysilsesquioxanes or silsesquioxane resins are straight-forward hydrolytic polycondensation reactions and typically do not require particularly precise control over the product structure regarding the molecular SSQs calling for long synthesis times, multistep procedures, various purification methods, or rigorous maintenance of reaction conditions, as discussed in detail by Cordes et al. [31]. Polysilsesquioxanes may be synthesized in water-based media and purified by water washing [38]. The work of Shimizu et al. [39] presents the synthesis of aerogels, for which the hydrolysis and condensation reactions were performed with ethyltrimethoxysilane (ETMS), vinyltrimethoxysilane (VTMS), methyltrimethoxysilane (MTMS), and tetramethoxysilane (TMOS) in the presence of nitric acid. They can be further functionalized by a hydrosilylation reaction with compounds having divinyl moieties [6].

#### 3.2.4. Post-Functionalization of POSS Cage

The methods of modification of silsesquioxanes systems can be divided into two main groups: catalytic and non-catalytic reactions. The catalytic reactions include mainly hydrosilylation of alkene systems [28], which is commonly used in industry but also silylative coupling [40], and cross-metathesis [41], Heck coupling [42], Friedel–Crafts reaction [43], thiol-ene reaction [44], nucleophilic reactions [31], or even thermal dehydrative condensation [45]. The most common reactions for the post-functionalization of silsesquioxanes from the above mentioned are nucleophilic reactions and catalytic hydrosilylation. Nucleophilic reactions can be divided into nucleophilic substitution and nucleophilic addition. The first one is most commonly a substitution of chlorine or iodine atom with various organic groups [42,46,47,48]. Nucleophilic addition is commonly used in oxirane chemistry [49] and anhydride and amide chemistry [50]. Additionally, the alkyne–azide click reaction is a special kind of nucleophilic addition [51]. 

**Figure 6:**
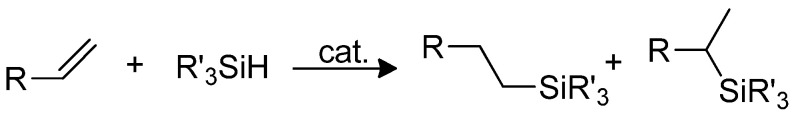
General scheme of hydrosilylation reaction.

The hydrosilylation reaction is one of the most basic and most frequently used methods of catalytic modification of silsesquioxanes (Figure 6). The reaction is based on the addition of compounds with Si-H groups to carbon–carbon, carbon–heteroatom, and heteroatom–heteroatom multiple bonds. The most commonly used are catalysts from the group of transition metals such as platinum, rhodium, palladium, nickel, iridium, cobalt, and iron [52]. 

## 4. Literature Review on Patent Analysis Regarding Silsesquioxanes in Cosmetics

The number of patents and cosmetic products containing POSS derivatives has grown intensively in recent years. The earliest reports in the field of patent protection concerning the use of silsesquioxanes in cosmetics date back to the late 1960s and early 1970s. In those years, the companies General Electric (GE) and Dow Corning (DOW) [53] were the leaders in the application of organosilicon compounds. At that time, the first applications of phenyl silsesquioxanes with a ladder structure in the preparation of emulsions were presented [54]. Until the end of the 1970s, references to the use of silsesquioxane compounds in the proprietary formulations may be considered marginal. The 1980s also showed that the use of SSQs in cosmetic products was associated with quality benefits. There are reports related to their use, for example, in non-liquid makeup cosmetics [55] but there was still no clear interest in these compounds in the cosmetics industry. In the early 1990s, a definite change in this trend was observed. The first patent applications of the French company L’Oréal appeared [56]. However, the DOW and Japanese inventors still played a leading role. In the 90s, protection was applied mainly to the use of polymethylsilsesquioxanes in emulsions [57] and systems containing minerals with the addition of polydimethylsilsesquioxanes [58]. The advantages of SSQs with perfluorinated groups in hair care products have also been noticed [59]. In the 90s, the dominant applications of SSQs were in hair preparations, varnishes, and makeup systems with mineral fillers. A noticeable increase in the interest in international patent protection for cosmetics with the use of silsesquioxanes occurred at the end of the 20th century. In the years 2000–2010, relatively many reports of the use of SSQs in microencapsulated formulations were found [60]. In recent years (2015–2020), there has been an increase in interests in sunscreen formulations [61]. Since the middle of the first decade of the 21st century, the areas of application of SSQs have significantly expanded: hair styling preparations [62], w/o (water in oil) emulsifiers [63], and UV protection systems [64]. When referring to the analysis of patent applications, it should be borne in mind that silsesquioxanes play the role of one of many ingredients in complex systems of cosmetic products. For this reason, many researchers convey a superficial impression of the multifunctionality and multitude of applications of POSS compounds. Based on detailed analyzes of the content of the applications, it seems that silsesquioxanes play a similar role to functional polysiloxanes; however, due to the higher equivalent of functional groups per mass unit and the condensed, non-linear nature of their structure, they differ from polysiloxane analogs. In a great number of applications, SSQs are present in parallel with polysiloxanes [65] or are applied as silisesquioxane/polysiloxane crosspolymers [66]. Therefore, it cannot be said that SSQs simply act as highly concentrated functional substitutes for silicone oils; rather, they complement and extend their functionality in cosmetic formulations, for instance, based on different physio-chemical characteristics. From the beginning of the second decade of the 21st century, an increasing patent activity has been observed in the People’s Republic of China [62,63,66,67,68]. The described applications of SSQs are becoming more and more advanced in terms of their functionality in cosmetic preparations. It was suggested to use SSQ in makeup systems for the treatment of mineral compounds (TiO_2_) in order to obtain specific optical properties related to light scattering [69]. It should be noted that the increase in the advancement and complexity of patent applications in the last decade does not result from the use of SSQs in cosmetics but the overall development of this industry. Due to low solubility, the loading of silsesquioxanes in cosmetic preparations ranges from 0.1% *w*/*w* to 2%. The silsesquioxanes play the role of a functional component, mainly operating in the interface, similarly to the organofunctional silanes in the polymer/filler systems. In many cases, their role is to create interactions between the polymer/copolymer systems and the water-containing emulsion systems [68].

**Figure 7:**
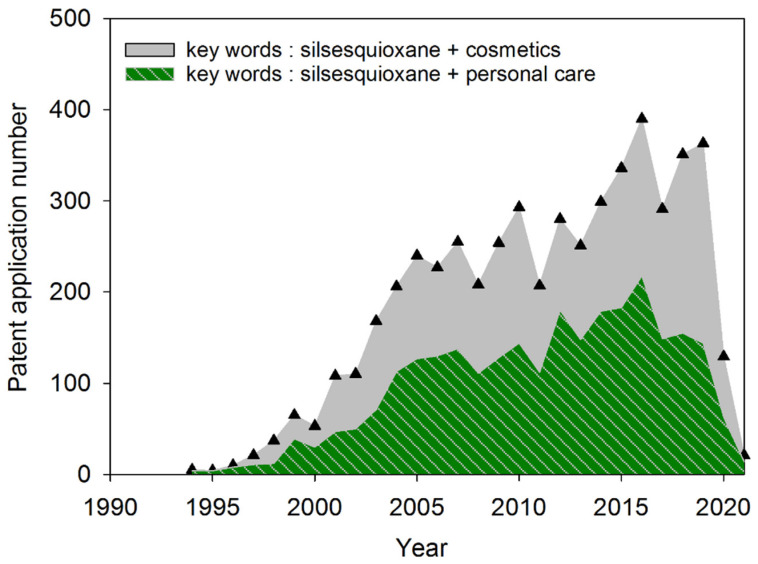
Several patent applications in the field of silsesquioxanes/cosmetics and silsesquioxanes/personal care (based on Google Patents database).

In summary, in the field of patent protection, the dominant structural forms are polysilisesquioxanes and polysilsesquioxane resins. These forms are much more common in cosmetic formulations than structurally pure polyhedral forms. Inventions subject to patent protection on leading international markets in the years 2000–2020 are dominated by the L’Oréal concern (nearly 30% of all patent applications), followed by Kose Corp. with approximately 8%, Henkel with 4%, and The Procter & Gamble Company with 3%. Other brands that used SSQ are Japanese concerns such as Shiseido Nippon Fine Chemical, Kao, and Sinopec. It is noticeable that the increase in the number of patent applications is rather linear, and in the years 2005–2020, it did not exceed 10–20% on an annual basis. The impact of the epidemic on the reduction in the number of reports in 2020–2021 is also clearly noticeable (Figure 7).

## 5. Application of POSS Derivatives in Cosmetic Products

Cosmetic industries are continuously searching for new compositions that will provide comfort, long-lasting effect, and natural-looking color. POSS have found applications in different cosmetic products that will be the subject of the below-presented subsections. In Table 1 examples of various derivatives of silsesquioxanes that are dedicated to personal care are shown.

### 5.1. Hair Care Products

Hair care compositions can be classified into two main categories:

- products that provide temporary effects such as shampoos, conditioners, and sprays
- products with long-lasting effects such as permanent waves and permanent colors [85].

POSS derivatives can be incorporated into hair care products such as shampoos, conditioners, and styling gels. POSS molecules substituted with one or more cation-containing groups can be applied in conditioners and hair care products. Furthermore, they can be used as styling agents as ionic attractive forces towards charged sites on strands of hair [17]. Due to adhesive features, POSS molecules can be applied to increase the long-wear properties of hair coloration. Another study proved that, when organosiloxanes (such as trialkoxy aminosilanes) were added to conditioner formulations, the volume of hair was increased [86]. A similar effect was observed when polymethylsilsesquioxane resins were introduced to hair care products [3]. Another example of POSS derivative that can be applied in hair care products is amino-functionalized silsesquioxane. It was reported that a water-soluble, 3-(2-aminoethyl)aminopropyl derivative provided curl retention for hair [86]. Silsesquioxanes have also found application as set-holding agents in hair fixative products [87]. They provide outstanding hold, higher humidity resistance, and form stiffer film compared to typical organic resins. It should be highlighted that silicones do not accumulate on the hair surface. They are washable with surfactants and do not burden the hair. Unlike vegetable oils, which burden hair, they do not leave a feeling of greasiness. Modern silicone conditioners ensure hair regeneration and facilitate styling. The film formed on the hair is light, flexible, and leaves a feeling of dry and silky touch.

### 5.2. Color Cosmetics

Today, the segment of color cosmetics is increasing among the customers. To this group of cosmetics belong products dedicated to facial make-up, eye make-up, and lip care. Apart from the visual aspects that are provided by these cosmetics, customers are searching for formulations that remained on the skin intact for long periods of time. The color cosmetics include inter alia liquid makeup and loose and pressed powders. Typically, the color cosmetics consist of pigments and waxes suspended in carrier oils as well as film-formers [2]. When the product is applied on the skin, the volatile compounds evaporate, and the film containing pigments and oils remain on the skin surface. The long-lasting features of pigments are dependent on the ability of film to adhere to the skin, on its mechanical properties, and on the cohesive strength of the dried cosmetic product. Silicones have been applied in the development of long-wear formulations since the 1990s [88]. So far, different wear technologies have been developed such as formulations consisting of, among others, solid pigments, organosiloxanes and diorganopolysiloxanes. However, these types of cosmetic products are tacky during and after their application. Moreover, the film that is formed on the skin causes uncomfortable sensations of tautness. In response to this need, the cosmetic compositions that contain polyhedral oligomeric silsequioxanes (POSS)-grafted polyolefins have been developed [89]. L’Oréal used these compounds in cosmetic formulations to preserve long-wear, decreased tackiness, and enriched color, while Avon obtained composition containing graftable POSS that formed non-transferable, long-lasting film that left a comfortable feeling on the skin or lips [90]. However, in recent years, much attention has been also paid to other advanced silicones that bring long-lasting benefits for skincare products. Apart from trimethylsiloxysilicate (MQ resins), silsesquioxane resins were also proposed to be used in long-wear cosmetics [2]. A mixture of two silicone resins such as trimethylsiloxysilicate and polypropylsilsesquioxane (Dow Corning MQ-1650 Flake Resin) is applied in color cosmetics and skin care products. The resins create the hard and brittle film. The application of silicones blend enhances the intensity and shine of color. Another POSS derivative that can be applied as additive ingredient in color cosmetics such as lipsticks is polyphenylsilsesquioxane. Additionally, alkyl phenyl silsesquioxane resisns were incorporated into lipsticks providing high gloss [72]. Moreover, POSS trisilanols can be used as dispersants for pigments [91]. Additionally, POSS compounds, due to their unique structure, can be applied to compatibilized different ingredients in skincare formulations. Various molecules such as dyes can be surrounded by the cage-like structure of POSS or can exist partly in/outside the POSS cage. MQ copolymers are applied to obtain color cosmetics [92]. It was also proposed to apply polypropylsilsesquoxane in the eyeshadow compositions [78]. The conventional eyeshadows produce a dry texture that is difficult to apply. Therefore, it is important to develop cosmetic products that will have soft textures and long-lasting effect. It is possible when polypropylsilsesquioxane is mixed together with a volatile solvent, silica, and boron nitride [78]. Combinations of POSS and non-POSS silicone resins in formulations improve wearing, pliability, and texture compared to silicone-resin containing products. This combination can be used in mascaras, lip coatings, eyeliners, body make-up, and nail coatings [93]. Silsesquioxanes have also found applications in the grinding of pigment. They ensure better uniformity of grind and consequently enhance pigment dispersibility [6]. 

### 5.3. Nail Care Products

On the market, there are different types of nail products available, such as nail polish (known as lacquers, varnish, and enamels), artificial nails (recognized as gels or acrylics), and nail polish remover [94]. The artificial nail formulations are applied not only to enhance the appearance of nails but also to strengthen their surfaces. As ingredients of nail polish products, SSQs can increase the strength and durability of the finished coating. SSQs substituted with epoxide or thiol groups can form a covalently bond film and, therefore, can have better adhesion to the nail than enamel. Examples of POSS derivatives that can be applied in nail care products:

- MercaptopropylIsooctyl-POSS
- EpoxyCyclohexylCyclopentyl-POSS
- EpoxyCyclohexylCyclohexyl-POSS
- EpoxyCyclohexylIsobutyl-POSS
- GlycidylIsooctyl-POSS
- Octaepoxy-POSS.

Acryloyloxylpropyl polysilsesquioxane is used as a nail conditioning agent [79]. It is obtained by the hydrolysis and condensation of acryloyloxy propyltrimethoxysilane. It consists of the mixture of 3D siloxane polymers and oligomers with a partial cage structure. Ethyl polysilsesquioxane and isobutyl polysilsesquioxane are other examples of compounds that can be applied as a nail conditioning agent.

Polyhedral oligomeric silsesquioxanes are also applied in formulations for nail coating. Typical nail coating consists of three different layers such as a base (increases adhesion between the nail and the other layers), a color, and a topcoat (ameliorates durability). However, it is of great interest to reduce the number of layers in order to accelerate and facilitate its applications process. The incorporation of POSS to the nail coating products solves this problem. The presence of POSS in the color layer ameliorates adhesion to the nail and, therefore, a base layer is not required. Furthermore, due to polyhedral oligomeric silsesquioxanes, the hardness and gloss of the cured nail coating is improved [95]. The examples of compounds that can be used in the nail coating are POSS derivatives where at least one R group are polyethylene glycol units and other groups are alkyl units. Depending on the type of personal care formulation, different amount of the POSS can be used. The amount of POSS applied in nail polish and other cosmetics ranges from about 0.005% to 40% by weight of the final product.

### 5.4. Skincare Products

The main challenge for cosmetic formulators is to obtain stable and efficient skincare products such as emulsions. Generally, they can be classified into single emulsions (such as water-in-oil and oil-in-water) and multiple emulsions (such as oil-in-water-in-oil and water-in-oil-in-water). They consist of two or more immiscible liquid phases [96]. One phase is dispersed into droplets in another continuous phase. Typically, two liquid phases have various surface tensions; therefore, the emulsions are thermodynamically unstable. It should be added that there are also water-in-silicone (w/s) emulsions [97], in which water droplets are dispersed in silicone. They consist of the water phase, silicone oil, and w/s emulsifiers (silicone polyether) [98]. The main advantage of this system is that it can be obtained at room temperature, and it has a high-water resistance [99]. The most important factor is to obtain homogeneous formulations that do not undergo any destabilization processes such as creaming, flocculation, coagulation, coalescence, phase inversion, or Oswald ripening [100,101,102]. In order to reduce the surface tension between two liquid phases, it is required to apply emulsifiers that are used to improve emulsion’s stability by increasing the kinetic energy [103]. Recent studies have proven that amphiphilic POSS derivatives can be used as potential emulsifiers [104,105]. Imoto et al. [106] obtained tripodal amphiphilic POSS derivatives that contain hydrophilic poly(ethylene glycol) tails and hydrophobic centers. The synthesized compound was studied as a potential emulsifier during emulsion preparation and was also compared with mono-substituted amphiphilic POSS derivatives. In order to assess the potential of POSS derivatives as emulsifiers, their 1 wt% aqueous solutions were prepared. Afterwards, these solutions were homogenized with methyl myristate. Additionally, the same procedure was repeated in the absence of POSS derivatives, and no emulsion was obtained. These results indicated that functionalized polyhedral oligomeric silsesquioxanes affected the stability of the emulsion. The diameter of the emulsion droplets of the formulation with POSS derivative remained almost the same size after 1 month of storage. This proved that tripodal polyhedral oligomeric silsesquioxanes can serve as an effective emulsifier to obtain a highly stable emulsion. 

**Figure 8:**
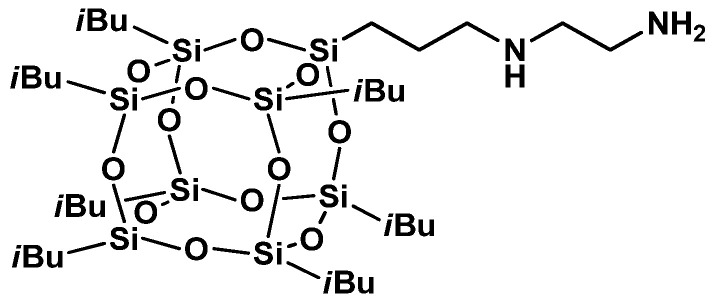
Aminoethylaminopropylheptaisobutyl POSS molecular structure [108].

Recently highly concentrated emulsions have attracted attention due to their useful applications in different fields such as pharmaceutics, food products, and cosmetics. However, the main drawback of these systems is their instability during their processing and storage [107]. The instability results from uncontrolled coalescence that has an influence on the properties of the final formulation. Masalova et al. reported that POSS nanomolecules can be used to stabilize highly concentrated w/o emulsions (HCE) [108]. It was proven that 3-(2-aminoethyl)aminopropylheptaisobutyl POSS (Figure 8) exhibited the ability to decrease interfacial tension and had a high capacity to obtain HCE. These features were not observed when conventional surfactants were applied. 

Furthermore, it should be added that silsesquioxanes also found application in anti-aging products. Moreover, this compound was proposed to hide and fill in the wrinkles [109]. It should be added that polymethylsilsesquioxane is composed of particles with a median size from 0.5 to 15 μm, and, therefore, it can be easily distributed in different formulations.

The excessive exposure to sunlight can cause serious skin problems including melanoma [110]. Therefore, it is recommended to apply sunscreen cosmetics to protect the human body. There are two types of sunscreen ingredients—chemical and physical [111]. The first one absorbs UV radiation and the second one scatters ultraviolet rays [112]. Cinnamic acid, benzophenone, and salicylic acid are examples of chemical sunscreens, while titanium dioxide and zinc oxide belong to the group of physical filters [113,114]. The main drawback of chemical sunscreens is that some of them can be absorbed into the skin, causing irritation and allergic reactions. On the other hand, physical sunscreens leave a white film after application that is uncomfortable for users [115,116]. Therefore, it is important to develop new types of UV filters. Silsesquioxanes as hybridized organic–inorganic materials can be an appropriate candidate for a sunscreen agent. The functional groups capable of providing UV protection can be attached to the POSS structure. These substituents can be bound to the cage of POSS either directly or by using the following bridging molecules—azo, diazo, epoxy, olefin, or halogen-containing compounds, by appropriate reactions of addition or substitution.

SSQs such as poly(p-methoxycinnamaminyl)propylsilsesquioxane and poly(p- ethoxycinnamamidyl)propylsilsesquioxane) that can be applied as UV-absorbing ingredients have been obtained by Kim et al. [117]. The synthesized compounds were then added to petrolatum, and the sun protection factor was assessed. The measured SPF values were notably higher than formulations containing other tested filters. Furthermore, no white spots were observed on the skin when the composition with POSS derivatives was applied. The results proved that both POSS compounds are promising UV-absorbing agents in cosmetic formulations. Another important advantage of silsesquioxanes is that they can form water-resistant films in sun care products [3].

Another patent presents the fact that polysilsesquioxane spherical particles can be applied in sunscreen formulations [118]. First, the silsesquioxane precursors with ultraviolet absorbers were synthesized, and, next, polysilsesquioxane spherical particles were obtained. The as-prepared compound was introduced to the cosmetic composition, and its effectiveness was checked. The results proved that formulations containing polysilsesquioxane spherical particles provide UV protection and exhibit less whitening phenomenon compared to traditional sunscreen formulations. Nanoparticles of bridged polysilsesquioxane were also applied in sunscreens. The photodegradation level and leaching was found to be minimized. The formulations based on the bridged polysilsesquioxanes had broad-spectrum photoprotective ability [119]. It should be highlighted that, due to the addition of polymethylsilsesquioxane to sun protection products, their application is very soft and smooth. Furthermore, polymethylsilsesquioxane exhibits oil absorption properties that decrease the greasy skin-feeling derived from some of the sunscreen blockers. 

Hydrogels are classified as 3D polymeric networks that are able to absorb large amount of water [120]. The crosslinking nodes can be created by covalent bonds or by different physical interactions such host–guest complexation, hydrogen bonding, or ionic association. The hydrogels found applications in various fields including biomaterials, biotechnology, and pharmacy [121]. However, it should be stressed that they exhibit poor stability and mechanical properties [122]. In order to solve these problems, nanocomposites hydrogels can be used. Furthermore, polyhedral oligomeric silsesquioxanes can be introduced to hydrogels to improve both chemical and physical features [123]. The results proved that POSS-containing hydrogels exhibit better mechanical properties compared to conventional hydrogels. POSS molecules tend to self-aggregate in aqueous solution due to their hydrophobicity. Additionally, they can act as physical crosslinkers in polymer networks [124] and thus increase the mechanical strength of hydrogels. Octa-methacrylate POSS, octa-vinyl POSS, and octa(glycidoxypropyl) POSS can be also applied as crosslinker in hydrogels [125]. Mather et al. obtained silver-containing hydrogels using POSS diol, PEG, and lysine methyl-ester diisocyanate dedicated to wound healing. The formulation gave a prolonged antimicrobial effect that prevented from infections [126].

## 6. Future Outlooks

The future in the cosmetic industry is to introduce new (macro)molecules for specific applications in order to fill the voids of present personal care formulations, based on the experience of the cosmetics manufacturers and the opinions of the end-users. The customers provide feedback on the performance of the products or the lack thereof, while the specialists in the field of designing and manufacturing of those products can link the reported issues or shortcomings of a given formula to the selection and balance of the chosen ingredients comprising the formulation. On the basis of this knowledge, the new compounds of appropriate physicochemical properties, such as rheology, temperature-dependent physical state, or emulsifying properties, may be tailored to provide solutions to flaws of present formulations. The new compounds should be economical in industrial manufacturing and easily formulated into cosmetic products designed for customers’ satisfaction, as well as avoid the concerns of the environmental burden caused by their release into the natural environment. While molecular silsesquioxanes may not become a preferable choice for cosmetic formulations anytime soon due to their high cost of production, polysilsesquioxanes and various hybrids thereof, such as polysilsesquioxane-siloxane copolymers or resins, may provide a satisfactory choice for both high-performing and cost-effective solutions, especially when comprising one of the special-purpose additives among multi-component ingredients used for manufacturing of the final product.

## 7. Conclusions

The earliest reports in the use of silsesquioxanes in cosmetics date back to the late 1960s and early 1970s. However, it was not until mid 1990s that a boom was observed in the industry. It was caused by both the development of the cosmetics industry itself and the growth of importance of this field in the global economy, as well as the intensive development of research on SSQs influence in complex systems containing polymers, water-based media, emulsions, etc., which resulted in the increase in the real value of their applications. The combination of the economic factor with the rapid development of research in this area led to the actual use of this group of compounds in cosmetic industry. It should be clearly emphasized that there is a significant advantage of polysilsesquioxanes structures in practical applications, as reported in several articles and patents providing data on several parameters such as color stability, film stability, coating strength, UV protection, and more. This is due to the large difference in production costs between disordered structures and molecular compounds. The challenges of the future in research on the use of silsesquioxanes in the cosmetics industry are modeling functional properties, reducing synthesis costs, and designing multifunctional polysilsesquioxanes.

## Figures and Tables

**Table 1:** POSS dedicated to cosmetic products.

International Nomenclature of Cosmetic Ingredients (INCI)	Commercial Name	Producer	Cosmetic Product Category	Functions	Refs.
Polymethylsilsesquioxane	SilForm*Flexible Resin	Momentive Performance Materials	color cosmetics, creams, lotions, sun care	opacifying agent, film former, provide long-lasitng wearability	[2,70]
	BELSIL^®^ B 110	Wacker Chemie AG	skin care, sunscreen	providing softness, enhanced spreading	[71]
	BRB PMS-2	BRB International BV	color cosmetics, skin care	pigment stabilizer	[72]
	BRB PMS-5	BRB International BV	color cosmetics, skin care	pigment stabilizer	[73]
	BELSIL^®^ SPR 45 VP	Wacker Chemie AG	color cosmetics, skin care, sunscreen	providing softness, water repellence, film former	[74]
Blend of cyclopentasiloxane and polypropylsilsesquioxane	DOWSIL 670 Fluid	Dow	color cosmetics, suncare, skin care	film former, provide long-lasting color, shine enhancer	[2,75]
Polysilsesquioxane Steardimonium Chloride	SiQube^TM^ Q1850	Paradigm Science Inc.	skincare, haircare	cleansing ability, skin barrier function improver	[76]
Blend of MQ (trimethylsiloxysilicate and T propyl (polypropylsilsesquioxane) silicone resins	MQ-1640 Flake Resin	Dow Corning	color cosmetics, skin care, hair care	provide hair long lasting colour	[77]
Isobutyl Polysilsesquioxane	PSS-Octaisobutyl substituted 97	Boc Sciences	nail care	nail conditioning agent	[78]
Acryloyloxypropyl Polysilsesquioxane	SLT-3R01	Gelest	nail care	nail conditioning agent	[79]
Trimethylpentyl Polysilsesquioxane	Isooctyl POSS	Hybrid Plastics	nail care	nail conditioning agent	[79,80]
Epoxycyclohexylethyl Polysilsesquioxane	EP0408	Hybrid Plastics	nail care	nail conditioning agent	[81]
Glycidoxypropyl Polysilsesquioxane	EP0408	Hybrid Plastics	nail care	film forming ability	[82,83]
Methoxy PEG-10 Polysilsesquioxane	PG1190	Hybrid Plastics	skin care	skin conditioning agent, cleansing agent, solubilizing agent	[79,83,84]
